# Levels of Evidence in Small Animal Dentistry and Oral Surgery Literature Over 40 Years

**DOI:** 10.3389/fvets.2020.00454

**Published:** 2020-07-31

**Authors:** Lindsey A. Schneider, Patrick C. Carney, Erin R. B. Eldermire, Nadine Fiani, Santiago Peralta

**Affiliations:** ^1^Department of Clinical Sciences, College of Veterinary Medicine, Cornell University, Ithaca, NY, United States; ^2^Flower-Sprecher Veterinary Library, Cornell University, Ithaca, NY, United States

**Keywords:** levels of evidence, evidence-based medicine, study design, dentistry, oral surgery, veterinary research

## Abstract

Veterinary dentistry and oral surgery are relatively new clinical disciplines that have rapidly evolved in the last few decades. Although clinical standards of care are supported by a growing body of literature, the extent to which peer-reviewed, evidence-based studies have contributed to advancing the practice of dentistry and oral surgery has not been assessed. The purpose of this study was to survey literature on the clinical practice of small animal dentistry and oral surgery published over the past 40 years to evaluate the levels of evidence over time, authorship affiliation, funding, and clinical subdisciplines within the field. A literature search was conducted in PubMed and the identified articles were screened for inclusion. A total of 1,083 articles were included for final analysis. Three reviewers independently assessed and assigned each article to one of nine predetermined study design categories. Systematic reviews and meta-analyses were considered the highest level of evidence, whereas expert opinion and experimental (*ex vivo, in vitro*, or *in silico*) studies were deemed the lowest levels of evidence. For statistical analysis and interpretation, study type was dichotomized into high evidence designs from which causal inference and/or associations could be derived, and low evidence designs which were purely descriptive or non-clinical experiments. No statistically significant difference in the distribution of study type was seen over time, with the majority of research in the last 5 years being largely at high risk of bias and descriptive in nature: 80.6% of articles published between 2014 and 2019 were assigned to the low evidence design tier. The type of study was found to differ by author affiliation: high evidence study designs were found more often than expected when author affiliation was multi-institutional or industrial, whereas private practice authorship was underrepresented in the high evidence design tier. To meet the increasing demand for evidence-based studies on the practice of dentistry and oral surgery in dogs and cats, researchers are encouraged to consider study design when testing hypotheses to improve the quality of research.

## Introduction

As any medical discipline emerges and evolves, the standards of care also evolve with the growth of the body of evidence, represented by published clinical experiences, and research. Veterinary dentistry arose as a unique clinical discipline largely as the result of interest and expertise developed by veterinarians in small animal practice. The American Veterinary Dental College (AVDC) was established in 1988 and was followed a decade later by the formation of the European Veterinary Dental College. Also in 1988, the *Journal of Veterinary Dentistry* began publication and remained the only specialty journal for veterinary dentistry until 2015, when the specialty section of “Veterinary Dentistry and Oromaxillofacial Surgery” was added to *Frontiers in Veterinary Science*. In 2019, fourteen AVDC Diplomates were recognized as Founding Fellows in Oral and Maxillofacial Surgery. The past four decades have thus seen the rapid development of small animal dentistry and oral surgery. As these disciplines continue to expand in scope and in the number of practitioners, it is important to assess the published literature to examine where there are gaps in knowledge and to understand where clinicians and researchers can best direct their efforts to build an evidence-based foundation for clinical practice.

The clinical practice of veterinary dentistry and oral surgery—or any medical discipline—involves frequent decision-making based on the available evidence, but not all published clinical evidence is of the same quality. The representativeness of the study population and funding bias are two examples of factors that influence the quality of reported information. Hierarchical frameworks structured on potential factors influencing the validity of evidence are a foundational construct of evidence-based medicine (EBM), and they help clinicians critically evaluate the quality of research to make informed decisions (1, 2). Various hierarchies or systems have been described, from a report by the Canadian Task Force on the Periodic Health Examination in 1979 to the Center for Evidence-Based Medicine's *Levels of Evidence for Therapeutic Studies* (3, 4). These hierarchies rank studies according to the probability of bias. General consensus places systematic review or meta-analysis of randomized controlled trials at the highest level and case series/reports and expert opinions at the lowest levels. Systematic reviews methodically synthesize the results of multiple studies, while controlled trials have less risk of containing systematic errors because they are designed to minimize bias, usually through design elements like blinding, randomization, and placebo control. Expert opinion, in contrast, is potentially biased by the author's experiences and heuristics, while lacking a formal process to evaluate the validity of the opinion. Case-control, cohort, and cross-sectional studies are intermediate within hierarchies of evidence because these study designs permit statistical comparisons between groups and have well-established methodologies to minimize bias, but lack of randomization and potential difficulties in ascertaining outcomes and exposures increase the risk of bias relative to higher-tier designs. Conversely, case reports and case series report descriptive data only, so they are considered lower level evidence. Finally, experimental *ex vivo, in vitro*, or *in silico* studies can contribute substantially to the practice of medicine, but because pathology may be induced rather than naturally occurring and reported outcomes may not be clinical, it can be difficult to relate the findings directly to everyday medical decisions. The role of basic science research is difficult to compartmentalize within hierarchies of evidence, and justifications can be made to place this study type into either high- or low-evidence categories. Understanding scientific mechanisms is not sufficient for therapeutic decision making, particularly when other kinds of evidence are available, so basic science research is conventionally considered low evidence.

Within several disciplines in both human and veterinary medicine, levels of evidence have been considered and assessed. For example, several articles on evidence-based medicine have been published in plastic and reconstructive surgery journals (5–7), pediatric orthopedics (8), and veterinary surgery (9, 10) and the American Society of Plastic Surgeons has adopted a series of evidence rating scales to guide clinicians in assessing the quality of published results (11). Some categorization systems are limited by the type of research questions allowed: studies comparing treatment outcomes are inherently different than studies investigating prognosis, diagnosis, or economic analysis. Therefore, a robust way of categorizing studies to determine their level of evidence had to be developed.

This study sought to characterize the body of literature on the practice of small animal dentistry by testing the hypotheses that the fewest number of studies are in the highest level of evidence and the most studies in the lowest level, but that the proportion of studies in the high evidence study design tier has been trending up over time. Furthermore, we sought to determine whether specific subdisciplines contain a higher number of publications and higher levels of evidence than others, and whether authorship affiliation or funding source were correlated with the study designs of published research.

## Materials and Methods

### Literature Search

A systematic literature search was performed on articles published between January 1, 1980 and January 28, 2019 for manuscripts relating to veterinary dentistry and oral surgery. The specific strategy for the search was developed by a research librarian with expertise in evidence synthesis (EE) (Supplementary Table 1). The search strategy incorporated both general search terms as well as Medical Subject Headings (MeSH terms) from the National Library of Medicine's controlled vocabulary thesaurus used for indexing articles in the electronic database PubMed. Eighteen journals listed in the AVDC's *Suggested Reading List for Candidates and Trainees* were identified as the most relevant publications. Ulrich's Periodicals Directory was consulted to verify that all journals in the AVDC-suggested reading list were indexed in PubMed, and they were incorporated into the search strategy, although the search was not limited to these journals.

A hand search of the *Journal of Veterinary Dentistry* was also performed to complement the electronic database search because that publication was expected to yield a high proportion of relevant articles. During acquisition of the manuscripts identified in the PubMed search, it was noted that not all relevant articles from the *Journal of Veterinary Dentistry* were captured within the database search, so the table of contents of every issue available online (those published between 1999 and 2019) were reviewed, and articles that met the inclusion criteria were then added to the search results. The results from the PubMed database search as well as the hand search were imported into reference management software (EndNote X8, Clarivate Analytics 2017) for additional screening and data collection. The EndNote software was used to find and eliminate duplicate references.

### Literature Screening and Data Collection

Inclusion and exclusion criteria for study selection were predetermined to reduce bias. After obtaining the search results, articles were collected and further screened for inclusion based on the following criteria: the study addressed an issue related to the clinical practice of veterinary dentistry and oral surgery; the study was limited to the domestic dog or cat; full text and/or full-length abstracts were available through the Cornell University Library; and the study was published in English. Studies were excluded if they reported duplicate data or if disease was experimentally induced or treatment was performed for the purpose of human research models with a low possibility of translation into clinical veterinary practice (e.g., implant dentistry).

One reviewer (LS) independently screened all search results by title and abstract according to the aforementioned inclusion criteria. The full-text versions of the included articles were obtained and uploaded into EndNote. Because only one reviewer performed the initial screening for inclusion, any questionable articles were included for screening by two additional reviewers (NF, SP), and the article was excluded after review of the full manuscript if at least two reviewers deemed the article met the exclusion criteria. Articles meeting inclusion criteria were independently reviewed and assigned a study design by three reviewers (LS, NF, SP).

Nine categories of study design were established that roughly followed a hierarchy of levels of evidence: systematic review/meta-analysis; clinical trials; cohort studies; case-control studies; cross-sectional studies; case series; case reports; expert opinion; and experimental *ex vivo*/*in vitro*/*in silico* studies. To keep reviewers blinded to one another, all data collection was performed independent of other reviewers. A random number generator was used to randomize the order of the articles for screening to minimize any learning bias. Reviewers were given access to the EndNote library containing the full-text articles and recorded data (study design classification) on separate spreadsheets (Excel 15.33, Microsoft Office, Redmond, WA 2016). Reviewers met after screening 100 articles to discuss the data collection methodology and refine their criteria for assigning study design category. The spreadsheets for each of the three reviewers were subsequently merged for final data tabulation (Supplementary Table 2).

One reviewer (LS) collected data on the subdiscipline within dentistry and oral surgery; authorship affiliation; and funding source. Subdisciplines were grouped into ten categories as follows: diagnostic imaging; anatomy/development; periodontics; endodontics; prosthodontics; orthodontics; oral tumors; cranio-maxillofacial trauma; other oral medicine; and other maxillofacial surgery. Although many articles bridged multiple subdisciplines, for the purposes of data analysis, a single most relevant category was identified and recorded for each article. Authorship affiliation was categorized as academic institution, private practice, industry, or undetermined based on the published address of the first author. If the article explicitly stated that cases were enrolled from multiple institutions, then the authorship affiliation was characterized as multi-center. Finally, data on funding was collected by reviewing each included article for a declaration or acknowledgment of financial support. The funding source was categorized as public, private, both, or none/undetermined.

Following the completion of data collection by the three reviewers, agreement between study design assignment was assessed. Articles that were assigned the same category of study design by at least two reviewers were classified according to the majority. Articles with complete disagreement between the three reviewers were classified based on discussion and consensus by all three reviewers after the initial data collection.

### Data Analysis

Descriptive statistics were performed using Excel software. All additional statistics were performed in SAS 9.4 (SAS Institute, Cary, NC). The proportions of studies in each subdiscipline, study design, authorship affiliation, and funding source were tabulated. Agreement between reviewers' assignment of study design was assessed using an unweighted Cohen's kappa coefficient and associated 95% confidence interval (CI), with an interpretation paradigm as follows: poor (<0.20), fair (0.21–0.40), moderate (0.41–0.60), good (0.61–0.80), and very good (0.81–1.00). The kappa statistic is a measure of inter-rater reliability for ordinal or nominal data, where a value of 1.00 indicates that the reviewers agree in their classification of every item, and a value of 0.00 indicates agreement no better than that expected by chance. The kappa coefficient does not differentiate whether disagreement is due to random differences (i.e., those due to chance) or systematic differences (i.e., those due to a consistent pattern) between the individuals' ratings (12). An unweighted kappa coefficient tests for agreement or disagreement as a binary function and can be used for nominal or ordinal data, whereas a weighted kappa coefficient takes into account the degree of disagreement and can only be used for ordinal data. Unweighted kappa coefficients were reported because, while the scale used to rate studies was designed to be ordinal, reasonable arguments could be made for ordering the scale in a different manner; an unweighted kappa is agnostic with respect to the order, whereas a weighted kappa would change based on the ordering of categories. Although not reported, weighted kappa coefficients were calculated for each 2-way statistic and were all within 2% of the unweighted values.

The studies were grouped by half-decade increments and the Kruskal–Wallis test was performed to assess differences in the distribution of study types between half-decades. Chi-square testing was used to compare the distribution of study types between subdisciplines and authorship affiliation. To avoid zero cells and to avoid a large number of cells that might hinder *post-hoc* interpretation, study type was dichotomized into those designs from which causal inference and/or associations could be derived (systematic review/meta-analysis, clinical trials, cohort studies, case-control studies, and cross-sectional studies, referred to as the high evidence study design tier), and those which were purely descriptive or non-clinical experimental designs (case series, case reports, expert opinion, and experimental studies, the low evidence study design tier). For *post-hoc* assessment of statistically significant Chi-square results, adjusted standardized residuals were calculated, with absolute values >3 for a given cell indicating poor fit with the null hypothesis (13, 14). To compare the distribution of study type by funding source, the analysis was performed with study type as both the native variable and the dichotomized variable as described above. *Post-hoc* analysis was conducted as described above.

## Results

Through electronic database and hand searches, 2,902 studies were identified for screening, and 50.9% of those articles (*n* = 1,478) were excluded based on the aforementioned criteria after the title and abstract were reviewed by one reviewer. An additional 9.1% of the initially identified articles (*n* = 265) were excluded following appraisal by all three reviewers, and 2.6% of articles (*n* = 76) were excluded because full text or a full-length abstract were not available. In total, 1,083 articles published in 91 different journals were reviewed and underwent data collection (Table 1).

**Table 1:** Literature search and screening methodology.

Identification	Articles identified through database searching	*n* = 2,839	Duplicates removed *n* = 7
	Articles identified through hand searching	*n* = 70	
Screening	Titles and abstracts screened	*n* = 2,902	Articles excluded through screening *n* = 1,478 Articles excluded due to unavailability of full-text or full-length abstracts *n* = 76
Eligibility	Full-text articles or full-length abstracts assessed for eligibility by three reviewers	*n* = 1,348	Articles excluded on the basis of critical appraisal *n* = 265
Included	Studies included in quantitative synthesis	*n* = 1,083	

**Figure 1:**
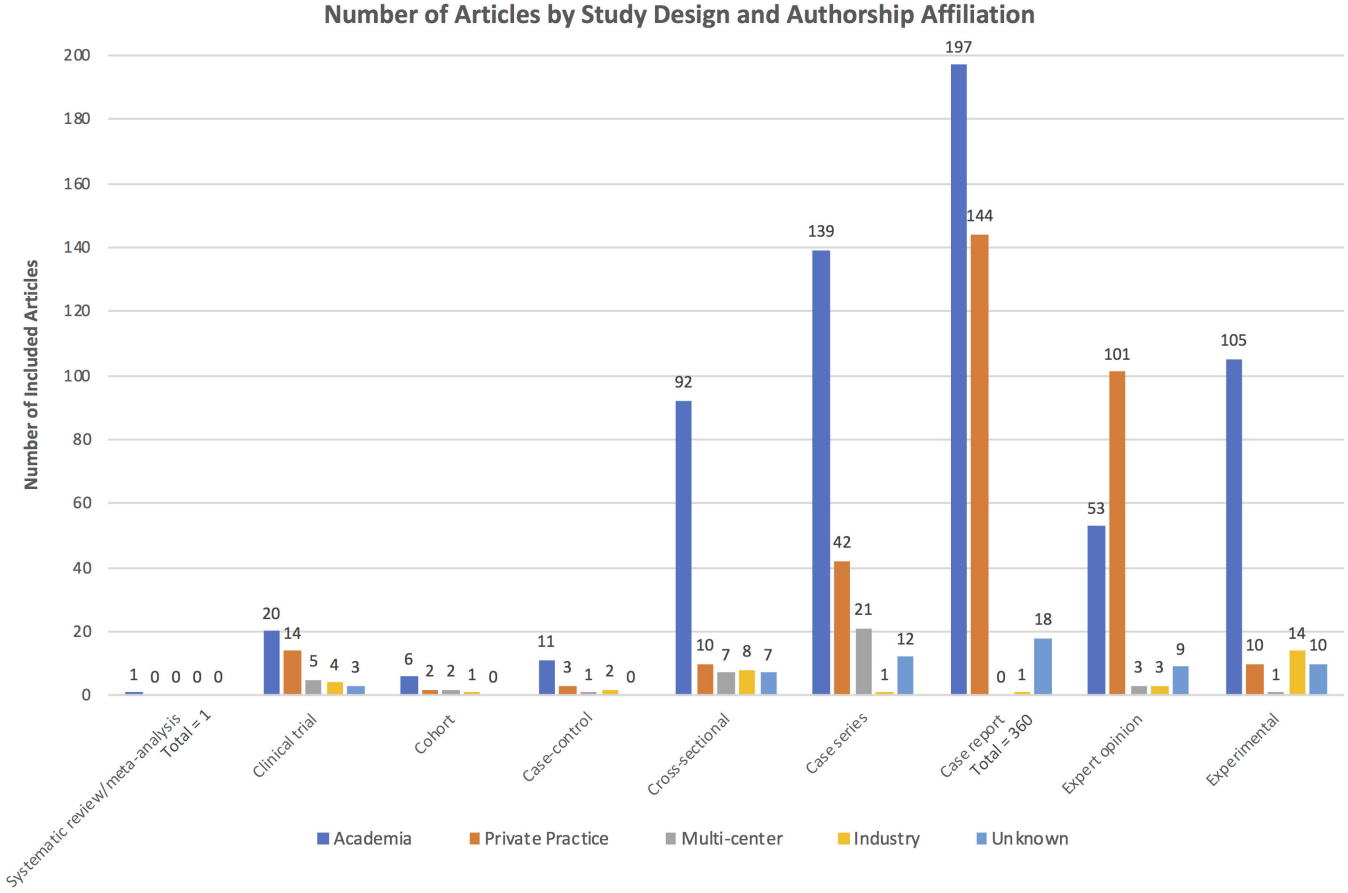
Distribution of publications by study design category. Colors demonstrate the authorship composition for each study design category.

The total number of publications in each study design category, as well as the authorship composition for each category, is shown in Figure 1. Case reports were the most frequent article type, comprising 33.2% of the included articles, whereas systematic review/meta-analysis was the least frequent, with only one article (<0.1% of the total). When dichotomized into two design tiers, the high evidence category was comprised of 199 studies representing 18.4% of included articles, while the remaining 884 articles (81.6%) were in the low evidence design tier.

Cohen's kappa statistic assessing inter-rater reliability of article classification found agreement between reviewers to be good: for reviewers 1 and 2, 0.73 (95% CI 0.70–0.76); for reviewers 1 and 3, 0.75 (0.73–0.78); and for reviewers 2 and 3, 0.76 (0.73–0.78). The overall (three-way) kappa coefficient was 0.75.

**Figure 2:**
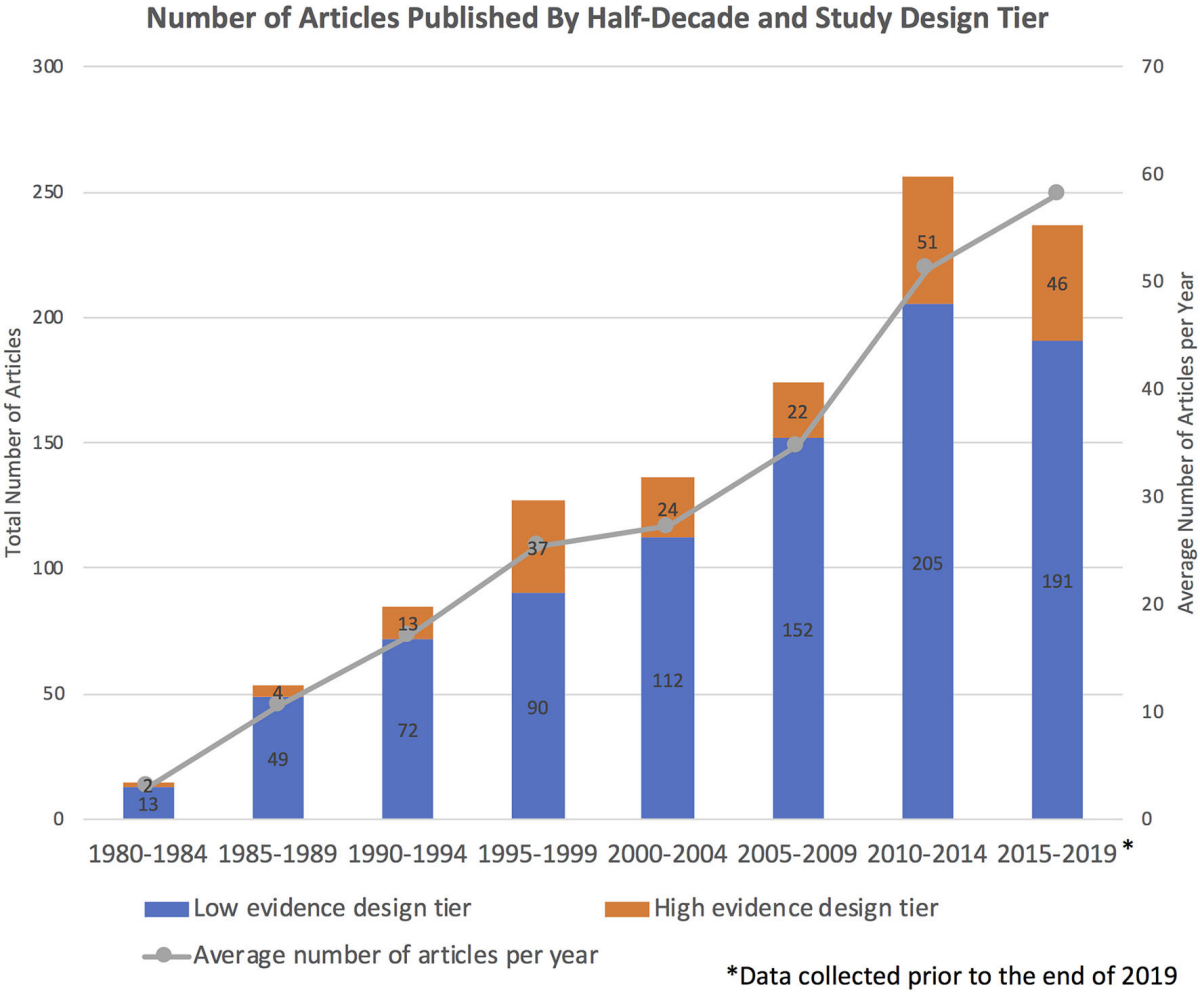
Number of articles published per half-decade, with colors depicting the proportion of articles in the high vs. low evidence design tiers. The line graph illustrates the near-linear growth in the average number of articles published per year. Note that 2015–2019 does not include a full 5 years because the literature search was performed prior to the end of 2019.

**Figure 3:**
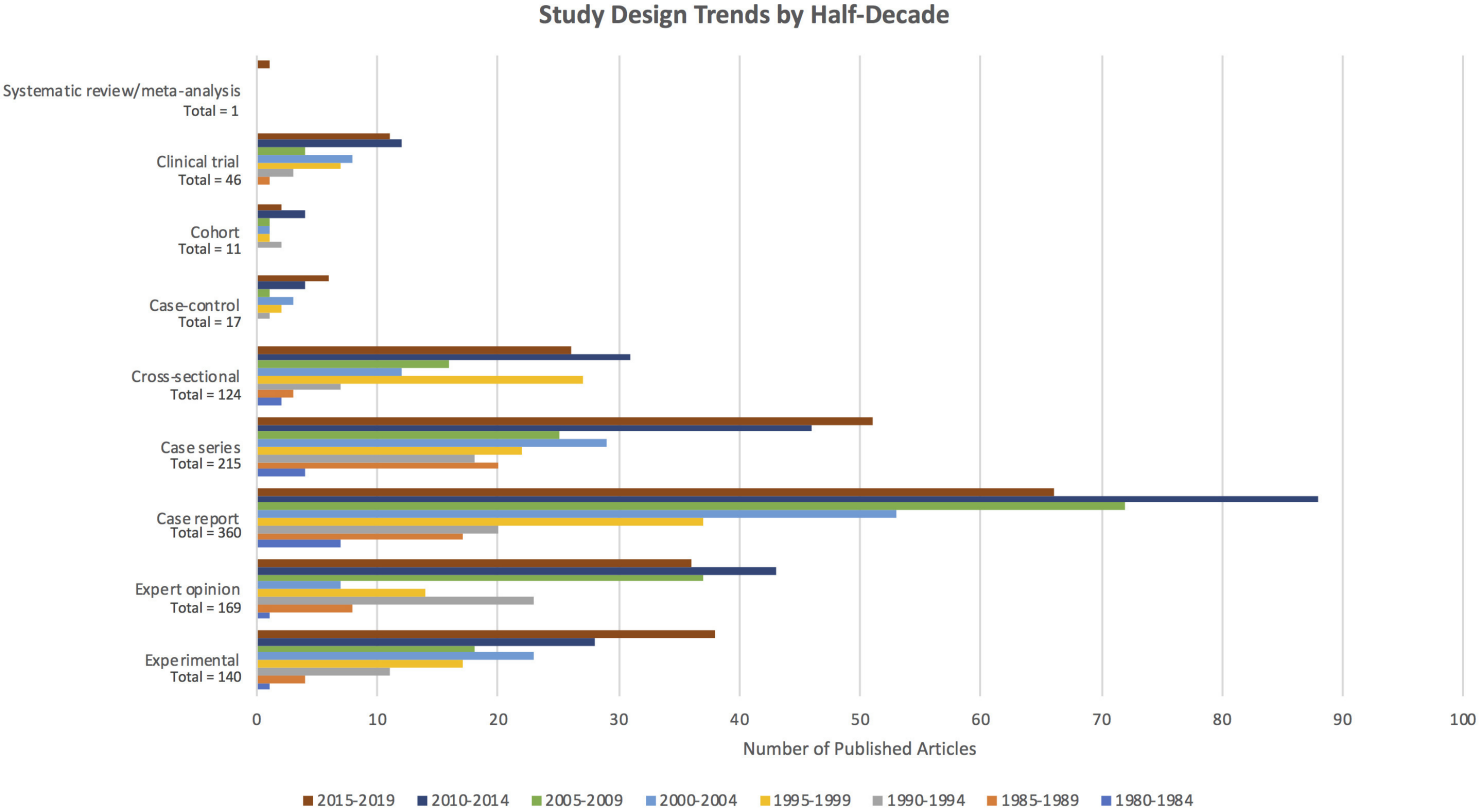
Number of articles published per study design category, with colors depicting the proportion of articles within each study design category published in each half-decade.

The number of articles published in each half-decade steadily increased, with near-linear growth in the average number of articles published per year (Figure 2). Fifteen included articles were published in the first half-decade (1980–1984), compared to 256 articles in 2010–2014. While the absolute number of high evidence tier articles published increased over time, no statistically significant difference in the distribution of study type was seen over time (Kruskal–Wallis *p* = 0.0914). For example, 80.6% of articles published between 2015 and 2019 were in the low evidence design tier, compared to 86.7% of articles published between 1980 and 1984. However, the number of published clinical trials increased from zero in the first half-decade (1980–1984) to 11 in the most recent half-decade (2015–2019). Trends in the number of articles published in each study design category by half-decade are depicted in Figure 3.

**Figure 4:**
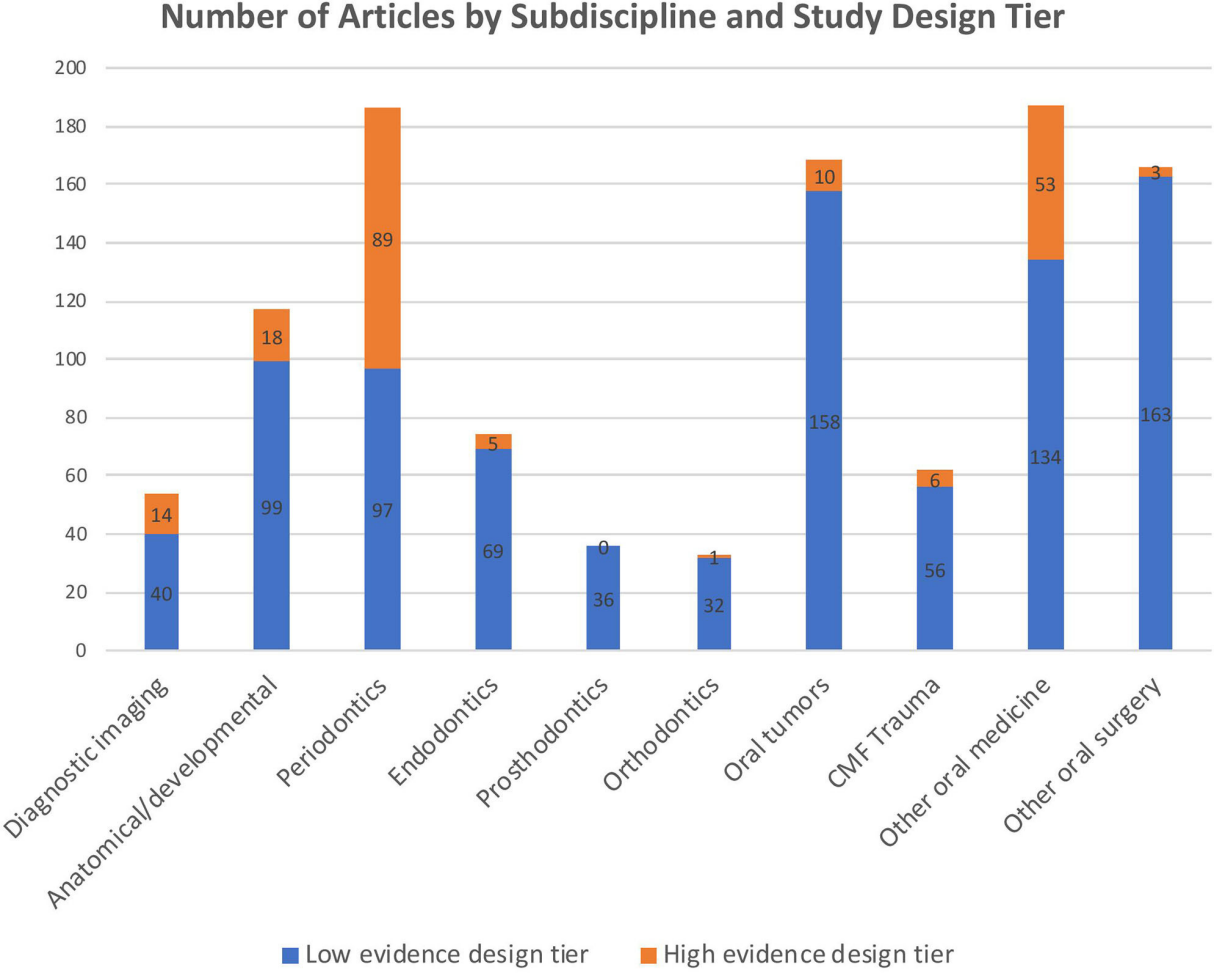
Number of articles published per subdiscipline, with colors depicting the proportion of articles in the high vs. low evidence design tiers.

The number of articles published in each subdiscipline, as well as the proportions of high vs. low evidence design tier articles within individual subdisciplines, are depicted in Figure 4. The type of study was found to differ by subdiscipline (Chi-square omnibus *p* < 0.0001). *Post-hoc* evaluation based on adjusted standardized residuals found the greatest difference to be in periodontics, with the high evidence design tier being more common than expected (adjusted standardized residual 11.49). A similar, although less dramatic, effect was seen for the category “other oral medicine” (adjusted standardized residual 3.89). Conversely, in the subdisciplines of “other maxillofacial surgery” (adjusted standardized residual −5.99) and “oral tumors,” the low evidence tier of study designs was represented more than expected.

The majority of articles (*n* = 624, 57.6%) were produced by academia, whereas multi-center studies accounted for only 3.7% (*n* = 40), and industry accounted for 3.1% (*n* = 34). Private practice produced 30.1% (*n* = 326) of the included articles, and authorship affiliation could not be determined for the remaining 5.4% (*n* = 59). The type of study was found to differ by author affiliation (Chi-square omnibus *p* < 0.0001). *Post-hoc* evaluation based on adjusted standardized residuals found that high evidence study designs were found more often than expected when author affiliation was multi-institutional (adjusted standardized residual 3.19) or industrial (adjusted standardized residual 3.94), whereas private practice authorship was underrepresented in the high evidence study design tier (adjusted standardized residual −5.29).

A funding source was not declared for 84.1% of articles (*n* = 911), while 11.3% (*n* = 122) were privately funded and the remaining 4.6% (*n* = 50) were funded by government. The type of study when assessed as a dichotomous variable was found to differ by funding source (Chi-square omnibus *p* < 0.0001), with private funding being overrepresented in the high evidence design tier (adjusted standardized residual 10.81), and with undetermined/no funding being underrepresented in the high evidence design tier (adjusted standardized residual −10.31). When study design was assessed as the native variable, studies with undetermined/no funding were substantially underrepresented in clinical trials (adjusted standardized residual −6.42), cross-sectional studies (adjusted standardized residual −7.19), and experimental studies (adjusted standardized residual −6.23), while being overrepresented in case reports (adjusted standardized residual 7.78) and expert opinion (adjusted standardized residual 5.69). Those studies with public funding were overrepresented in the experimental study design category (adjusted standardized residual 3.94) and were underrepresented in case reports (adjusted standardized residual −3.23). Studies with private funding were overrepresented in clinical trials (adjusted standardized residual 7.26), cross-sectional studies (adjusted standardized residual 7.78), and experimental studies (adjusted standardized residual 4.53), but were underrepresented in case reports (adjusted standardized residual −6.85) and expert opinion (adjusted standardized residual −4.82).

## Discussion

Evidence-based medicine, which entails merging individual experience with weighted scientific evidence, has become the gold standard for the clinical practice of most disciplines. Small animal veterinary dentistry and oral surgery are no exception, so an investigation into their evidence base within peer-reviewed publications was warranted. Evaluating trends in the levels of evidence of published research enables assessment of the utilization of EBM principles. The results of this study demonstrate that the body of literature on veterinary dentistry and oral surgery is steadily increasing, but the overall levels of evidence have remained proportionally relatively unchanged over the past 40 years.

In many cases, a basic change in study design will advance the level of evidence of the research. Case series and case reports are important for hypothesis generation and they play a role within surgical disciplines to highlight serious complications with a specific intervention. To produce higher-level research within the specialties of veterinary dentistry and oral surgery, however, authors must go beyond publishing descriptions of clinical experience and include a comparison group in the study. Furthermore, high-volume procedures are more amenable to study designs that provide a higher level of evidence. Some subdisciplines within veterinary dentistry and oral surgery lend themselves better to different types of research.

Previous studies in human medicine have shown that surgical research lags behind internal medicine with respect to the application of more sophisticated study designs for the study of clinical questions (5). Although it is unknown whether this trend also is present in veterinary medicine, the results herein demonstrated that the “other oral surgery” subdiscipline was underrepresented while “other oral medicine” was overrepresented in the high evidence design tier. Veterinary surgical disciplines have the same limitations as human surgical disciplines: ethical concerns about sham surgery, individual variations in surgical procedures that cannot be controlled for, and inter-surgeon variability that introduces bias. Surgery is considered by some to be an operator-dependent intervention, making randomization and blinding challenging and impractical. Therefore, results from multi-center surgical trials are generally more robust than results from smaller single-center or single-surgeon studies. Only 3.7% of the studies over the past 40 years in veterinary dentistry and oral surgery had multi-center authorship affiliations, so more diverse collaborations are encouraged to help improve study design as well as the quality of evidence.

Periodontics and other oral medicine were the subdisciplines with the most publications, and these were also the two subdisciplines that were overrepresented in the high evidence design tier. Periodontal disease, feline tooth resorption, and feline stomatitis (the latter two conditions being the subject of many articles in the “other oral medicine” subdiscipline) are arguably the most chronic and costly diseases to treat within small animal veterinary dentistry, so it is unsurprising that the most research is done in these fields. Overall, the composition of the body of literature on small animal veterinary dentistry and oral surgery fairly accurately reflects the relevance of certain diseases and subspecialties: the fewest publications were within the subdisciplines of orthodontics and prosthodontics, and these are the least commonly practiced subdisciplines. Endodontics represented a relatively small proportion (6.8%) of the publications, but human endodontics is a large field that is closely applicable to small animal endodontics, so the evidence base for this subdiscipline is not fully captured in an assessment of publications limited to cats and dogs.

The correlations identified between study type and funding are expected: clinical trials and experimental studies are typically prospective and costly to implement, so funding is often essential, and these two study design types were underrepresented in the undetermined/no funding category. Conversely, case reports and expert opinion require little to no financial investment, and they were overrepresented in the undetermined/no funding category. Funding opportunities and the trainee effect (i.e., trainees lack the time and funding to conduct high level-of-evidence studies) are significant barriers to study design development. There is relatively little incentive in the private sector to conduct research, and researchers with predominantly clinical training are at a disadvantage in the grant application process compared to Ph.D.-trained researchers. Also, clinicians in private practice may not have access to statisticians as readily as those in academia, so the literature published from private practice may be less robust in study design. Multi-institutional studies that include authorship from academia and private practice could potentially offer wide patient recruitment while also taking advantage of funding sources in academia.

The methodology of this study was similar to that used in various assessments of the levels of evidence in other medical disciplines. Literature searches in other studies have ranged from a single journal at discrete time points to multiple databases with no publication date limitations (5, 7–9, 15, 16). Because there is no “gold-standard” literature search, it is impossible to know the sensitivity and specificity of this sample of small animal dentistry and oral surgery publications. Identifying articles for inclusion was a limitation of this study. By restricting inclusion to articles that were available electronically, the data is likely biased toward more recent publications. However, articles unavailable online may not be widely accessible to veterinary practitioners, and therefore they do not comprise a significant portion of clinical decision making. Furthermore, a hand search was only conducted for the single journal expected to yield the highest proportion of relevant articles, so it is probable that some articles from other journals that would meet the inclusion criteria were not identified in the literature search. The published literature itself may be biased by failure to publish studies with negative results. To maximize the number of articles included in this cross-sectional review of the literature on small animal dentistry and oral surgery, all types of research questions (e.g., diagnosis, prognosis, and treatment) were considered. Editorial decisions of specific journals also likely played a role in the published levels of evidence. Some journals have series (e.g., “Diagnostic Imaging in Veterinary Dental Practice” in the *Journal of the American Veterinary Medical Association*, “Step-by-Step” and “Pathways” in the *Journal of Veterinary Dentistry*) whose format limits the study design, and these series increase the quantity of studies in the low evidence study design tier.

Another aspect of the study methodology that was carefully considered was how publications were assigned to a study design—and consequently level of evidence—category. Subjectivity was minimized by having three reviewers blinded to the others' assignments. Additionally, the order in which articles were assessed was randomized to reduce learning bias. Statistical analysis based on kappa coefficients revealed good agreement between reviewers, which was consistent with findings from another study that investigated intra-observer reliability when assigning study type and level of evidence (8). Although collecting data on subdiscipline was not novel (7), to the authors' knowledge, this was the first study on EBM that also collected data on authorship affiliation and funding sources. Journal impact factor has been compared between levels of evidence in other EBM studies (15, 16). Future studies of the small animal dentistry and oral surgery literature could assess the correlation between citation rates or journal impact factor with levels of evidence.

The clinical bias of the study design ranking scale used herein may be considered a study limitation. The scale placed basic science research (studies conducted at the tissue, cellular, and molecular levels that did not involve feline or canine patients) in the lowest level, despite the robust scientific methodology and internal validity in the majority of these studies. Additionally, study design is not always an indication of study quality: a well-designed case-control study may have more validity than a poorly designed clinical trial. If all else is equal though, a well-designed randomized clinical trial has less risk of bias than a well-designed case-control or cohort study. Another limitation of this study was that only one reviewer screened the initial search results for inclusion; however, given the number of articles identified by the literature search and the labor-intensive screening process, the authors felt that the best compromise was to include any questionable articles for screening by three reviewers. Despite these shortcomings, this study has established a technique for surveying the literature which can be used for other clinical disciplines and for future studies within small animal dentistry and oral surgery, or to compare levels of evidence between medical and surgical disciplines within veterinary medicine.

In a relatively new specialty, obtaining high-quality evidence is challenging. The evolution of study design from case reports to controlled studies is an important indicator of the utilization of evidence-based medicine. The concept of EBM has been discussed over the last 40 years as the disciplines of small animal veterinary dentistry and oral surgery have evolved, yet its application in research has been underwhelming. By characterizing the study designs, subdisciplines, authorship affiliation, and funding sources within the body of literature on the practice of small animal dentistry and oral surgery, trends have been identified that can be used to incentivize the publication of higher levels of evidence.

## Conclusion

The small animal veterinary dentistry and oral surgery literature has been dominated by lower quality evidence over the last 40 years. Furthermore, private practice authorship affiliation and lack of funding appear to be barriers to the production of high evidence study designs. However, this study demonstrates a progressive increase in the absolute number of controlled studies (systematic review/meta-analysis, clinical trials, cohort studies, case-control studies, and cross-sectional studies). Some limitations to study design (e.g., number of subjects enrolled, ethical concerns with placebo treatment) are intrinsic to the field and cannot readily be changed. However, clinicians are encouraged to consider the levels of evidence in clinical decision-making. Given that strength of evidence was higher when authorship affiliation was multi-institutional or industrial and when private funding was available, researchers are encouraged to consider collaborations across academia, private practice, and industry to increase enrollment in studies and to take advantage of all available financial resources.

## Data Availability Statement

All datasets generated for this study are included in the article/Supplementary Material.

## Author Contributions

LS performed literature searches, screened articles for inclusion, performed data collection and descriptive statistics, and primarily authored the manuscript. PC helped establish the study design categories and performed statistical analyses. EE helped design and implement the literature search. NF and SP proposed the study concept, helped develop the methodology, and collected data. All authors contributed to manuscript revision, read, and approved the submitted version.

## Conflict of Interest

The authors declare that the research was conducted in the absence of any commercial or financial relationships that could be construed as a potential conflict of interest.
