# Supplementary material for: Levels of Evidence in Small Animal Dentistry and Oral Surgery Literature Over 40 Years
Source: Front Vet Sci. 2020 Jul 31;7:454. doi: 10.3389/fvets.2020.00454 (PMC7412963; doi:10.3389/fvets.2020.00454)
Supplement: Supplementary file 1 [file Table_1.DOCX]

## Supplementary Table 1.

Search Strategy for PubMed Database Performed on January 28, 2019

| **Search no.** | **Search sequence** | **Number of results** | **Notes** |
| --- | --- | --- | --- |
| #1 | (dentistry OR dental OR oral surgery OR jaw disease OR teeth OR tooth) | 920615 | General search for dentistry terms |
| #2 | "veterinary" [Subheading] OR "Veterinary Medicine"[Mesh] OR "Pathology, Veterinary"[Mesh] OR "Surgery, Veterinary"[Mesh] | 355089 | Searching veterinary terms using MeSH |
| #3 | #1 AND #2 | 4744 | Combining general dentistry terms with veterinary MeSH terms |
| #4 | ("Cats"[Mesh]) OR "Dogs"[Mesh] | 429943 | Searching cats and dogs using MeSH |
| #5 | (((Felis catus[tiab]) OR cat[tiab]) OR cats[tiab]) | 136726 | General search for cat terms |
| #6 | (((Canis familiaris[tiab]) OR dog[tiab]) OR dogs[tiab]) | 209084 | General search for dog terms |
| #7 | #4 OR #5 OR #6 | 503334 | Creating a comprehensive cat and dog search |
| #8 | #3 AND #7 | 2105 | Combining veterinary dentistry with cats and dogs |
| #9 | (Journal of Veterinary Dentistry[TA] OR Journal of Endodontics[TA] OR Journal of Periodontology[TA] OR Journal of Clinical Periodontology[TA] OR Journal of the American Dental Association[TA] OR American Journal of Dentistry[TA] OR Frontiers in Veterinary Science[TA] OR American Journal of Veterinary Research[TA] OR Journal of the American Animal Hospital Association[TA] OR Journal of the American Veterinary Medical Association[TA] OR Journal of Small Animal Practice[TA] OR Journal of Feline Medicine and Surgery[TA] OR Journal of Veterinary Internal Medicine[TA] OR Veterinary Comparative Orthopaedics and Traumatology[TA] OR Veterinary Pathology[TA] OR Veterinary Radiology and Ultrasound[TA] OR Veterinary and Comparative Oncology[TA] OR and Veterinary Surgery[TA]) | 113600 | Searching for 18 journals listed in the AVDC suggested reading list (excluding *Equid Dentistry*) |
| #10 | #1 AND #7 AND #9 | 2149 | Combining general dentistry terms with comprehensive cat and dog search and journals |
| #11 | #8 OR #10 | 3130 | Either veterinary dentistry with cats and dogs or general dentistry with cats and dogs in journals of interest |
| #12 | #11 AND Publication date [1/1/1980 – present] | 2834 | Limit to publication dates on or after 1/1/1980 |
